# Risk of multi-drug resistant *Campylobacter* spp. and residual antimicrobials at poultry farms and live bird markets in Bangladesh

**DOI:** 10.1186/s12879-020-05006-6

**Published:** 2020-04-15

**Authors:** Sucharit Basu Neogi, Md. Mehedul Islam, S. K. Shaheenur Islam, A. H. M. Taslima Akhter, Md. Mahmudul Hasan Sikder, Shinji Yamasaki, S. M. Lutful Kabir

**Affiliations:** 1 Graduate School of Life and Environmental Sciences, Osaka Prefecture University, Osaka, Japan; 2 Department of Microbiology and Hygiene, Bangladesh Agricultural University, Mymensingh, 2202 Bangladesh; 3 Epidemiology Unit, Department of Livestock Services, Krishi Khamar Sarak, Farmgate, Dhaka, 1215 Bangladesh; 4 FAO-Food Safety Program (FSP), Institute of Public Health, Mohakhali, Dhaka, 1215 Bangladesh; 5 Department of Pharmacology, Bangladesh Agricultural University, Mymensingh, 2202 Bangladesh

**Keywords:** *Campylobacter*, Poultry farm, Live bird market, Multi-drug resistance, Residual antimicrobial, Risk factors

## Abstract

**Background:**

Understanding potential risks of multi-drug resistant (MDR) pathogens from the booming poultry sector is a crucial public health concern. *Campylobacter* spp. are among the most important zoonotic pathogens associated with MDR infections in poultry and human. This study systematically examined potential risks and associated socio-environmental factors of MDR *Campylobacter* spp. in poultry farms and live bird markets (LBMs) of Bangladesh.

**Methods:**

Microbial culture and PCR-based methods were applied to examine the occurrence and MDR patterns of *Campylobacter* spp. in potential sources (*n* = 224) at 7 hatcheries, 9 broiler farms and 4 LBMs in three sub-districts. Antimicrobial residues in broiler meat and liver samples (*n* = 50) were detected by advanced chromatographic techniques. A questionnaire based cross-sectional survey was conducted on socio-environmental factors.

**Results:**

Overall, 32% (71/ 224) samples were found contaminated with *Campylobacter* spp. In poultry farms, *Campylobacter* spp. was primarily found in cloacal swab (21/49, 43%), followed by drinking water (8/24, 33%), and meat (8/28, 29%) samples of broilers. Remarkably, at LBMs, *Campylobacter* spp. was detected in higher prevalence (*p* < 0.05) in broiler meat (14/26, 54%), which could be related (*p* < 0.01) to bacterial contamination of drinking water (11/21, 52%) and floor (9/21, 43%). *Campylobacter* isolates, one from each of 71 positive samples, were differentiated into *Campylobacter jejuni* (66%) and *Campylobacter coli* (34%). Alarmingly, 49 and 42% strains of *C. jejuni* and *C. coli*, respectively, were observed as MDR, i.e., resistant to three or more antimicrobials, including, tetracycline, amoxicillin, streptomycin, fluoroquinolones, and macrolides. Residual antimicrobials (oxytetracycline, ciprofloxacin and enrofloxacin) were detected in majority of broiler liver (79%) and meat (62%) samples, among which 33 and 19%, respectively, had concentration above acceptable limit. Inadequate personal and environmental hygiene, unscrupulously use of antimicrobials, improper waste disposal, and lack of health surveillance were distinguishable risk factors, with local diversity and compound influences on MDR pathogens.

**Conclusion:**

Potential contamination sources and anthropogenic factors associated with the alarming occurrence of MDR *Campylobacter*, noted in this study, would aid in developing interventions to minimize the increasing risks of poultry-associated MDR pathogens under ‘One Health’ banner that includes poultry, human and environment perspectives.

## Background

*Campylobacter* spp. are among the leading causes of food-borne infections causing human gastroenteritis [1] in both developing and developed countries. Between 2 and 20 million cases per year of human campylobacteriosis has been reported to occur in the European Union [2]. Children under 5 years and adolescents aged 15 to 25 years are more susceptible to campylobacteriosis, while immune-compromised people can develop prolonged and severe illness by *Campylobacter* spp. [3]. The common clinical symptoms of campylobacteriosis include fever, abdominal pain, and diarrhea, which are mostly self-limiting, but antimicrobials, including erythromycin, tetracycline, aminoglycosides, and fluoroquinolones are often used, particularly, for immunocompromised patients [4, 5]. *Campylobacter* has been associated with at least 11–21% of diarrhea episodes in children from low income countries [6]. *Campylobacter* spp., including *Campylobacter jejuni*, are also recognized as antecedent cause of particular post-infectious neuropathies, namely, Guillain-Barré syndrome, and autoimmune diseases, including Miller Fisher syndrome, reactive arthritis and irritable bowel syndrome [7]. Gastroenteritis by *Campylobacter* infections showed an increasing trend since the last decade [8]. The cytolethal distending toxin (CDT), consisting of CdtA, CdtB, and CdtC subunits, of *Campylobacter* spp. is one of the major virulence factors which contributes to cell cycle arrest, leading to the apoptosis or necrosis of immune cells, and epithelial cells in the intestine [9].

The gastrointestinal tract of poultry, wild birds, and livestock animals constitute the primary reservoir of *Campylobacter* spp. *Campylobacter jejuni* and *Campylobacter coli* are the predominant species of *Campylobacter* in poultry [5, 10]. These bacteria are commonly known to be harmless gut flora of chicken, which remain healthy but serve as carriers for human infection. Nevertheless, *C. jejuni* infection can induce inflammatory response, damage to gut mucosa and diarrhea in not all but some flocks of broiler chicken in intensive poultry production system [11]. The majority (50 to 80%) cases of campylobacteriosis in humans is attributable to consumption of poultry products [12]. Ingestion of contaminated food, including edible products of poultry and livestock, dairy milk, and water is the major cause of campylobacteriosis in human. Community members, including children, may also become infected with *Campylobacter* spp. through direct contact of contaminated objects, particularly, as a consequence of poor sanitation and hygiene practice in developing countries including Bangladesh [6, 13].

Antimicrobial resistance, especially multi-drug resistance (MDR), has become a serious problem worldwide. Approximately 700,000 deaths have been estimated due to the resistant strains of pathogenic microbes in 2013 throughout the world, whereas, according to a prediction, ca. 10,000,000 people may die in 2050 if no action is taken [14]. Unwise antimicrobial use in poultry farms, and also their interactive environmental and industrial sources, e.g., agriculture and farmed or reared animals, and human populations, are inducing selective pressure to develop drug resistance among the zoonotic pathogens, including campylobacters [15]. Increasing occurrence of MDR pathogens, including *Campylobacter* spp., has been an alarming global issue for ‘One Health’, which consider the health of humans, animals, and the environment in a holistic manner [14, 16, 17]. *Campylobacter* strains showing resistance to multiple drugs, particularly, nalidixic acid, tetracycline, fluoroquinolones, and macrolides, have been isolated at increasing frequency among patients with gastroenteritis and diarrhea during the last decades in both the developed and developing countries [5, 17, 18]. In Bangladesh, a developing country, there have been an increasing Government efforts through registered veterinarians to promote a prudent use of antimicrobial agents and only for therapeutic purposes [19], yet these drugs are often applied indiscriminately in the poultry farms. Due to lack of hygienic practices and knowledge to manage rampant pollutions in different socio-environmental conditions, majority of the population in Bangladesh is highly prone to health disasters associated with MDR infections from the emergent poultry sector. Since a few studies have observed the occurrence and antibiotic susceptibility patterns of *Campylobacter* spp. in poultry farms, detection of antimicrobial residues, together with socio-environmental risks prevailing in poultry farming and LBMs in Bangladesh have not been adequately explored [20, 21] .

Being among the most heavily populated regions, there is a high demand of broiler meat and eggs to meet the protein need of the growing population in Bangladesh. Inevitably, the poultry industries in this country are not only increasing but also adopting intensive farming methods with frequent use of antimicrobials to achieve high production. This study aimed to detect *Campylobacter* spp., prevalence of antimicrobial resistance patterns, and the extent of antimicrobial residues in potential sources of poultry farms and LBMs, and explored the associated anthropogenic risk factors. The purpose was to empirically understand the risks of MDR infections by campylobacters, occurrence of antimicrobial residues and inducing socio-environmental factors along the poultry production and supply chain in Bangladesh.

## Methods

### Sampling sites, collection and processing of samples

**Fig. 1:**
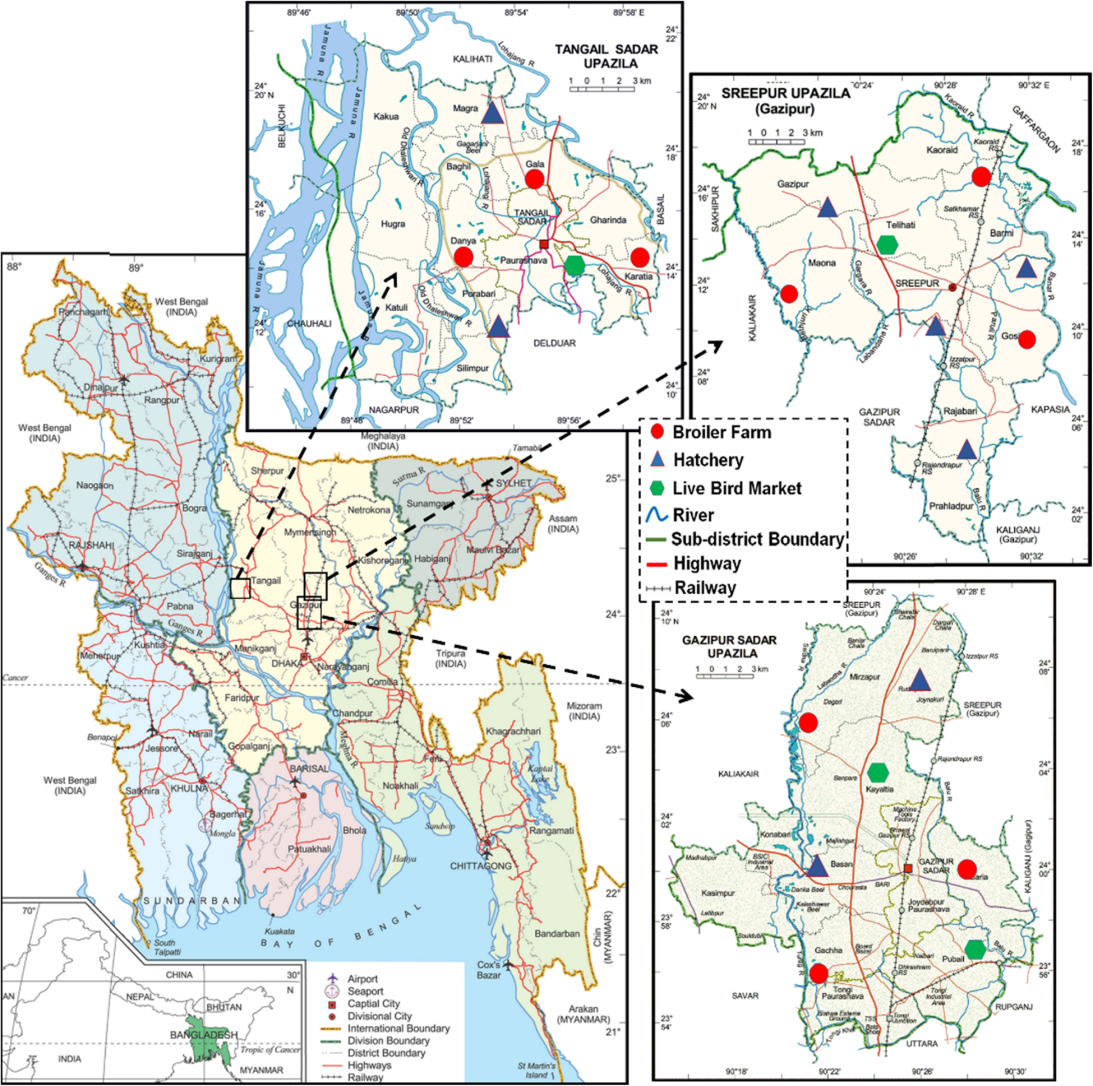
Map showing sampling sites in Bangladesh. Various kinds of samples were obtained from the selected hatcheries, broiler farms and live bird markets from three sub-districts or Upazilas (Tangail sadar, Gazipur Sadar and Sreepur) for detection of *Campylobacter* spp., and antimicrobial residues. The map was adapted from Banglapedia (https://www.banglapedia.org)

A total of 224 samples were collected during October, 2015 to May, 2016 from poultry sources, including 7 hatcheries, 9 broiler farms, and 4 LBMs in three sub-districts (Gazipur sadar, Sreepur and Tangail sadar) of Bangladesh (Fig. 1). Each of these sub-districts are among the regions with high number (> 3000) of poultry farms. The LBMs in these sub-districts are the primary commercial hub of trading poultry animals, which are mostly obtained from multiple farms at the nearby localities. The selection of the broiler farms was based on an inclusion criterion of a minimum flock size of 2000, while the hatcheries and LBMs located beyond 10 km^2^ of the selected broiler farms were excluded. In each sub-district, 3 broiler farms and at least 2 hatcheries, and one LBM were selected, keeping a minimum distance of 2 km for multiple sites of each category. Farm samples included broiler meats (BM-F, *n* = 28,), cloacal swabs (CS, *n* = 49), feed (F, *n* = 21), and drinking water (W, *n* = 24). Samples from hatcheries included chick meconium (CM, *n* = 33). LBM samples included broiler meats (BM-M, *n* = 27), drinking water (W, n = 21), and floor swabs (FS, n = 21) (Table 1). Aseptic measures were followed while collecting these samples, the amount of which varied type-wise: 100 g (wet weight) for meat and feed, 500 mL for water and 1–5 mL or mg swabbed materials in sterile cotton swab for faeces/cloaca and wet floor samples. The swab samples were preserved and transported in Cary-Blair transport media. Moreover, a total of 50 samples, including broiler meat (*n* = 26, each representing a composite of thigh, breast and drumstick) and liver (*n* = 24) were sampled from LBMs for screening of antimicrobial residues. To minimize sampling bias, three sub-samples of each sample were randomly collected and pooled together. The samples were immediately kept in sterile plastic containers, transported in an insulated foam box with cold chain (temperature, 4–6 °C) and processed within 6 h.

**Table 1:** Occurrence of *Campylobacter* spp. along the poultry supply chain in three sub-districts of Bangladesh*

Region	Hatchery		Broiler Farm (%)						Live Bird Market (%)				Total
(Sub-district)	CM (%)		F	W	CS	BM-F	Sub-total		W	FS	BM-M	Sub-total	(%)
Gazipur sadar	0/15 (0)		0/9 (0)	5/12 (42)	11/21 (52)	3/12 (25)	19/54 (35)		6/9 (67)	4/9 (44)	7/12 (58)	17/30 (57)	36/99 (36)
Sreepur	0/9 (0)		0/6 (0)	2/6 (33)	6/14 (43)	3/8 (38)	11/34 (32)		3/6 (50)	2/6 (33)	5/9 (56)	10/21 (48)	21/64 (33)
Tangail sadar	0/9 (0)		0/6 (0)	1/6 (17)	4/14 (29)	2/8 (25)	7/34 (21)		2/6 (33)	3/6 (50)	2/6 (33)	7/18 (39)	14/61 (23)
All	0/33 (0)		0/21 (0)	8/24 (33)	21/49 (43)	8/28 (29)	37/122 (30)		11/21 (52)	9/21 (43)	14/27 (52)	34/69 (49)	71/224 (32)
Significant	Association		Farm variables		Category (n)		*p*-value		Market variables		Category (n)		*p*-value
Relation					[+/+]: [+/−]	[−/+]: [−/−]					[+/+]: [+/−]	[−/+]: [−/−]	
(Fisher’s exact)			W / (CS + FBM)		[8]: [0]	[7]: [11]	0.0074	W / MBM			[10]: [3]	[1]: [7]	0.0075
			CS / FBM		[7]: [1]	[5]: [13]	0.0093	FS / MBM			[9]: [0]	[4]: [8]	0.0046
	Difference		Sub-district wise		Category (n)		p-value		Source wise		Category (n)		p-value
					[+]: [+]	[−]: [−]					[+]: [+]	[−]: [−]	
			Gazipur vs. Tangail		[36]: [14]	[63]: [47]	0.082	Farm vs. Market			[37]: [34]	[85]: [35]	0.0125
			(Farm & market total)					(Overall sub-total)					

*CM, F, W, CS, BM-F, FS and BM-M indicate chick meconium, feed, drinking water, cloacal swab, farm broiler meat, floor swab, and market broiler meat, respectively. Significant relations were determined by comparing data for each pair of cases under each category

### Culture based detection of *Campylobacter* spp.

The poultry-originated samples were screened for detection and isolation of *Campylobacter* spp. following filter-based selective culture method described by Shiramaru et al. (2012) [25]. In brief, the swab samples (1 g each) were suspended in sterile phosphate buffer saline (pH 7.4, 1 mL), while the meat samples (10 g each) were homogenized with the sterile saline (50 mL) using a tissue grinder (Seward Medical Ltd., London, UK). At least three replicates of a 100 μl portion of each suspended sample were spread onto a membrane filter (mixed cellulose ester type, 0.45 μm pore size, 47 mm diameter; Sartorius Stedim Biotech, Germany). In case of each water sample, a 100 mL portion in three replicates was filtered through the same kind of filter. Each of the membrane filters with inoculated samples was overlaid on blood agar medium (blood base agar no. 2; Oxoid, UK), supplemented with 5% defibrinated sheep blood, and kept at room temperature for 30 min. Afterwards, the filter was removed and the medium was incubated under microaerophilic conditions (5% O_2_, 10% CO_2_ and 85% N_2_) at 37 °C for 48 h. Suspected colonies were further cultured on blood agar plate in microaerophilic conditions stated above to obtain pure isolates. Presumptive *Campylobacter* isolates were subjected to species-specific morphological and biochemical assays, including Gram’s staining, motility test, catalase, oxidase, and hippurate hydrolysis, according to standard procedures [7].

### PCR-based confirmation of the species identity

DNA template of each isolate of *Campylobacter* spp. was prepared by boiling method described by Hoshino et al. (1998) [26]. In brief, a pure bacterial colony grown on blood based agar was mixed gently in 250 μL distilled water and subjected to boiling, followed by immediate cooling on ice, for 10 min each. The tubes were then centrifuged at 10,000X *g* for 10 min and the supernatant was collected as DNA template for polymerase chain reaction (PCR). Initially, PCR screening targeting 16S rRNA gene was performed according to Samosornsuk et al. (2007) [27] to confirm whether the strains belonged to the genus *Campylobacter*. Afterwards, *cdtC* gene based multiplex PCR was done for species-specific (*C. jejuni, C. coli* and *C. fetus*) identification following methods described by Asakura et al. (2008) [28]. DNA templates of *C. jejuni* ATCC33560, *C. coli* ATCC33559 and *C. fetus* ATCC27374 strains were used as positive controls, and that of *Escherichia coli* ATCC 25922 was used as a negative control. Details of all primers and corresponding PCR amplicon sizes are shown in Additional file 1. PCR products were subjected to gel electrophoresis (1.5% agarose, Invitrogen, USA) and after staining with ethidium bromide (0.5 μg ml^− 1^) and destaining with distilled water, each for 10 min, gel images were captured using a UV transilluminator (Biometra, Germany).

### Determination of antimicrobial resistance

All *Campylobacter* strains were tested for their resistance pattern by standard disk diffusion method. Eight commonly used antimicrobials with standard doses (μg) were examined: amoxicillin (AMX, 30 μg), azithromycin (AZM, 30), ciprofloxacin (CIP, 5 μg), erythromycin (ER, 30 μg), gentamicin (GM, 10 μg), tetracycline (TET, 30 μg), streptomycin (STR, 10 μg) and norfloxacin (NOR, 10 μg). In brief, freshly grown broth culture (equivalent to 0.5 McFarland turbidity) of each strain was uniformly inoculated, using sterile cotton swab, over the entire surface of Muller Hinton agar (Oxoid, UK), supplemented with 5% defibrinated sheep blood. Afterwards, 3–4 antimicrobial discs were placed in each agar plate and incubated at 37 **°**C for 48 h under microaerobic conditions (5% O_2_, 10% CO_2_ and 85% N_2_). The zones of growth inhibition were compared with the zone size interpretative standards as described by the Clinical and Laboratory Standard Institute (2016) [29] and thereby interpreted as susceptible (S), intermediate resistant (I) or resistant (R) to the antimicrobials*. E. coli* strain ATCC 25922 was used as a quality control organism. All data were confirmed by conducting at least two replicates of the disc diffusion experiments.

### Detection of antimicrobial residues

A total of 50 samples, including broiler meat (*n* = 26) and liver (*n* = 24) were tested for the presence of oxytetracycline, ciprofloxacin and enrofloxacin residues. Solid phase extraction of the samples was performed according to Popelka et al. (2005) [30]. A portion (4 g) of grinded meat or liver tissues was homogenized in 10 ml phosphate buffer (pH 6.5), and treated with 2 mL trichloroacetic acid (30%, v/v) to fractionate the proteins, followed by centrifugation at 7000X *g*, and sonication in an ultrasonic bath, 15 min each. The supernatant (ca. 2 ml) was treated with formaldehyde (100 μL) at 100 °C for 45 min, transferred to a new tube and mixed with equal amount of diethyl ether for 10 min at 25 °C, and the oily top layer was discarded. Extraction with diethyl ether was repeated twice. Afterwards, the extracts were filtered (0.45 μm), dried by evaporation and reconstituted with 2 mL mixture of 99% methanol and acetone (1:1) to obtain the final extract, which was stored at 4 °C.

Standard solutions of residues of three antimicrobial agents, namely, oxytetracycline, ciprofloxacin and enrofloxacin, each > 98% purity, were prepared by dissolving 1.0 g powder of each in 5 mL methanol (99%) and kept in the dark at 4 °C until analysis by thin layer chromatography (TLC) within a week following standard procedures [31]. Briefly, TLC silica plates of 0.25 mm thickness (Merck, Germany) were activated at 120 °C for 2 h before inoculating 50 μl of sample extract or standard solution of antimicrobial residues. Acetone-methanol (1:1) was used as mobile phase solvent and chromatographs were observed at 256 nm. Each of the samples was analyzed in triplicate. An internal standard and a blank was included after every five samples during analysis by both methods.

Quantification of residual antimicrobials were done by ultra-high performance liquid chromatography (UHPLC) for the meat and liver samples, which showed positive by TLC, following procedures described by Cooper et al. (1998) [32]. Stainless steel column Acclaim 120, C18 (5 μm, 120 Å, 4.6 X 250 mm) was used for chromatography (Dionex ultimate 3000 UHPLC). Phosphate buffer solution was prepared by adding di-sodium hydrogen phosphate to 0.2 M potassium di-hydrogen phosphate solution until pH 5.0. HPLC mobile phase constituted of distilled water and acetonitrile (85:15, v/v) for oxytetracycline, and 0.01 M phosphate buffer and acetonitrile (80:20, v/v) for others. Acetonitrile was HPLC grade (Panreac, Italy) and other reagents were of p.a. grade (Merk, Germany). A portion (25 μl) of each sample was injected into chromatographic column, equilibrated with mobile phase, and run at 0.8 ml min^− 1^ until the mobile phase ascended 7 cm. Afterwards, the column was air dried and visualized under UV light (λ = 254 nm and 366 nm). Standard controls (2 to 200 μg ml^− 1^) were prepared by serially (2-fold) diluting the stock solution of each antimicrobial. Six replicates of each concentration were assayed to standardize the regression equation (coefficient value > 0.99). Identification was done by comparing Rf values of antibiotic standards, i.e., 0.35, 0.80, and 0.97 for oxytetracycline, ciprofloxacine, and enrofloxacin, respectively. Peak area was used for antimicrobial quantification by regression analysis, Y = aX + b, where Y = component area or height, a = slope and b = intercept of the regression line, and X = estimated amount of antimicrobial. Extraction recovery was evaluated with comparison of peak areas for standard antimicrobial solution to that of the TLC-negative broiler tissue homogenates, spiked with the same standard solution.

### Data collection on health safety and hygiene practices

A total of 14 broiler farms, including the 9 sampled farms and additional 5 farms in the same geographical locations were enrolled for survey using semi-structured interviews through participatory methods to understand poultry husbandry associated risks, personal and environmental hygiene, and vulnerabilities to campylobacter infections, antimicrobial usages, and occupational safety. Each semi-structured interview was conducted using a questionnaire (see Additional file 2) and involving at least a couple of representatives from each of the selected farms (*n* = 14). Two teams (A and B), each comprising three experienced veterinarians from the Bangladesh Agricultural University, conducted the interviews at two phases upon prior written consent from each of the key informants, including farmer, farm manager or owner. Team A collected data from half of the selected farms (*n* = 7) in the first phase, which were verified by Team B in the second phase, and vice versa for the rest farms (n = 7). A total of 56 semi-structured interviews, including two from each farm at each of the two phases (3-month interval), were accomplished.

### Data management and statistical analyses

Data were recorded into Microsoft Excel 10 (Microsoft Corporation, Redmond, WA, USA) spreadsheet from the hard copies and statistically analysed using ‘Xact’ (ver. 7.21d, SciLab GmbH, St. Yrieix, France) and Statistica (ver. 10.0, StatSoft Inc., USA). Significant difference or association (*p* < 0.05) between the prevalence of *Campylobacter* species and any individual geo-socio-anthropogenic variables (study sites, and sample sources and types) was determined by Fisher’s exact test since the group-wise sample number was small. Antimicrobial susceptibility profile of the bacterial isolates was evaluated according to their differentiation into three independent groups, i.e., resistant, intermediate, and susceptible. MDR trait was defined as bacterial strains showing resistance against at least one antimicrobial agent in three or more antimicrobial classes [33]. Descriptive comparison of resistant patterns of *Campylobacter* strains with respect to diversity in sources and/or anthropogenic factors was performed using mean/ median and standard deviation, and also in the form of the box plots. Differences in the patterns and/or occurrence of antibiotic resistance in *Campylobacter* spp. (*C. coli* and *C. jejuni*) were calculated by the Paired Samples *t*-test. A ‘*p*’ value of < 0.05 was considered significant.

## Results

### Detection and occurrence of *Campylobacter* spp.

Of the 224 samples, a total of 71 (32%) were contaminated with *Campylobacter* spp. as determined by culture-based methods (Table 1). One representative colony of *Campylobacter* species was isolated in the form of pure culture from each of the 71 positive samples. When subjected to genus-specific 16S rRNA PCR, all of the isolated strains produced an expected amplicon size of 1530 bp, confirming their identity as *Campylobacter* spp. Approximately one-third (30%, 37 of 122) of the farm samples were observed to be contaminated with *Campylobacter* spp., with highest occurrence in cloacal swab (43%), followed by drinking water (33%) and broiler meat (29%) (Table 1). However, all of the chick meconium samples (*n* = 33) at hatcheries, and feed samples (*n* = 21) from the broiler farms were observed to be negative for *Campylobacter* spp. On the other hand, *Campylobacter* spp. was found in approximately half (49%, 34 of 69) of the LBM samples, including drinking water (52%), floor swab (43%) and broiler meat (52%). Spatial variation (sub-district wise) in the contamination rate was observed for all kinds of samples at both the farm and market sources, although statistical variation (*p* < 0.1, Fisher’s exact test) was only discernible between the total samples of Gazipur and Tangail sub-districts (Table 1). At the poultry farms, *Campylobacter* occurrence in cloacal swab and broiler meat samples had significant association (*p* = 0.0074, Fisher’s exact test). The risk of *Campylobacter* infection at broiler farms could be related to drinking water contamination, as indicated by its highly significant association (*p* = 0.0093) with the bacterial prevalence in broiler samples, both meat and cloacal swab (Table 1). Notably, the contamination rate for overall samples was significantly higher (*p* = 0.0125) at the LBMs when compared to the broiler farms. In the market environment, the observed high occurrence of *Campylobacter* spp. in broiler meat samples was associated with the bacterial relative occurrence in drinking water (*p* = 0.0075) and floor swab (*p* = 0.0043) samples (Table 1).

Multiplex PCR assay targeting the *cdtC* gene differentiated the *Campylobacter* isolates into 47 *C. jejuni* and 24 *C. coli*, producing species-specific amplicon of 524 bp and 313 bp, respectively (for representative gel images see Additional file 3). In both LBMs and farm environment, *Campylobacter* isolates were dominated by *C. jejuni.* Of the 37 isolates from the farm samples, 24 (65%) and 13 (35%) were identified as *C. jejuni* and *C. coli*, respectively. Similarly, out of 34 campylobacters isolated from the market samples, 23 (68%) and 11 (32%) were identified as *C. jejuni* and *C. coli*, respectively.

### Antimicrobial resistance patterns in *Campylobacter* strains

The zone of growth inhibition for each strain of *C. jejuni* and *C. coli* was compared with the interpretative standard [29] for each of the selected antimicrobials. Intermediate resistant isolates were considered as a group independent from those of resistant and susceptible isolates (see Additional file 4). Of the 47 *C. jejuni* isolates, majority showed resistant to amoxicillin (*n* = 30, 64%), erythromycin (*n* = 29, 62%) and tetracycline (*n* = 24, 51%), followed by ciprofloxacin (*n* = 17, 36%), norfloxacin (*n* = 12, 26%), streptomycin (n = 12, 26%), azithromycin (*n* = 7, 15%) and gentamicin (n = 2, 4%). Similarly, among the 24 *C. coli* strains a more frequent occurrence of resistance to amoxicillin (*n* = 13, 56%), erythromycin (*n* = 11, 46%) and tetracycline (*n* = 10, 42%), and comparatively less frequent occurrence of resistance to other tested antimicrobials, including ciprofloxacin (n = 7, 29%), norfloxacin (*n* = 8, 33%), streptomycin (*n* = 6, 25%), azithromycin (*n* = 4, 17%) and gentamicin (n = 2, 8%), were observed. In comparison to *C. coli*, a considerably higher number of *C. jejuni* strains were observed to be resistant to amoxicillin, tetracycline, erythromycin, and ciprofloxacin (see Additional file 4).

**Fig. 2:**
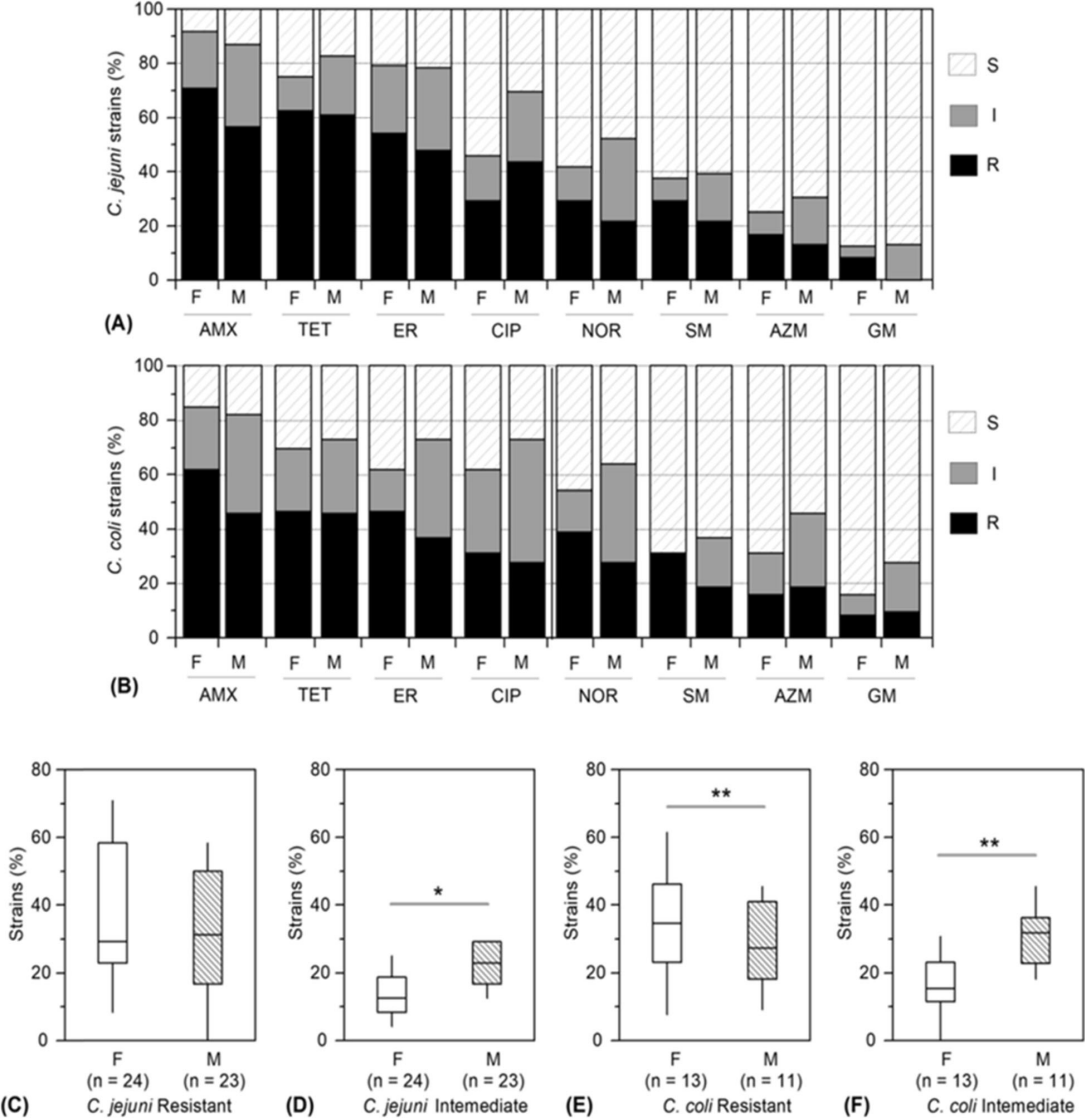
Source-wise comparative resistance traits in *C. jejuni* and *C. coli* strains. (A and B) Individual antimicrobial-wise comparison of the occurrence of resistance types among the strains of *C. jejuni* and *C. coli*, respectively, isolated from the farms (F) and live bird markets (M). Antimicrobial resistance types are indicated by S, I, and R, meaning sensitive, intermediate resistant and resistant, respectively. Eight commonly used antimicrobials at standard doses (μg) were examined: amoxicillin (AMX, 30 μg), tetracycline (TET, 30 μg), erythromycin (ER, 30 μg), ciprofloxacin (CIP, 5 μg), norfloxacin (NOR, 10 μg), streptomycin (STR, 10 μg), azithromycin (AZM, 30 μg), and gentamicin (GM, 10 μg). (C-D and E-F) Comparison between the farms and live bird markets of the variations in overall occurrence of full resistant or intermediate resistant strains of *C. jejuni* and *C. coli*, respectively. Composite variation of both resistance types in *C. jejuni* and *C. coli* strains, considering all the tested antimicrobials, are shown as box plots. The bottom and top of the box plots indicate the 25th and 75th percentile. Horizontal lines in the boxes indicate median values and their standard deviations are shown as vertical bars. *p < 0.05 and **p < 0.01, significant differences (paired *t* test) between mean values of the samples

A considerable portion of the *C. jejuni* and *C. coli* strains showed intermediate resistance pattern to the tested antimicrobials, ranging from 9 to 28% and 8 to 33% strains of these two species, respectively (Fig. 2 A-B, and Additional file 4). A higher percentage of *Campylobacter* strains isolated from farm than market samples were observed to be fully resistant to most of the tested antimicrobials, including amoxicillin, erythromycin, norfloxacin, streptomycin, and gentamicin. However, occurrence of intermediate resistance to the antimicrobials were observed to be significantly higher (*p* < 0.01) in *C. jejuni* and *C. coli* strains, isolated from LBMs, when compared to those of the farm samples (Fig. 2 C-F).

Among the 47 isolated strains of *C. jejuni*, 21% (*n* = 10) were resistant to two antimicrobial agents, while 23% (*n* = 11) were resistant to three antimicrobial agents. Alarmingly, about 20% (*n* = 9) strains of *C. jejuni* showed resistance against five or more antimicrobial agents, including amoxicillin, erythromycin, tetracycline, norfloxacin, azithromycin and gentamicin (Table 2). In case of 24 *C. coli* strains, 16% (*n* = 4) and 25% (*n* = 6) showed resistance against two and three antimicrobial agents, respectively, while a few, i.e., 4% (n = 1) and 12% (*n* = 3), were resistant to four and five antimicrobial agents (Table 2). Overall, *C. jejuni* strains showed higher occurrence (70%, *n* = 33) of resistance to at least two antimicrobial agents than that of *C. coli* strains (58%, *n* = 14). According to the modern perspective of MDR bacteria as those showing resistance against at least one antimicrobial in three or more antimicrobial classes [33], ca. 49 and 42% strains of *C. jejuni* and *C. coli*, respectively, could be defined as MDR.

**Table 2:** Occurrence of diversified resistance patterns among the poultry-originated *C. jejuni* and *C. coli* strains which showed full resistance against two or more antimicrobial agents

Resistance	Resistance patterns*	*C. jejuni* (*n* = 47)		*C. coli* (*n* = 24)	
Category		No. of strains (%)	Sub-total (%)	No. of strains (%)	Sub-total (%)
Against two	AMX-STR	3 (6)	10 (21)	2 (8)	4 (16)
	AMX-TET	5 (11)		2 (8)	
	ER-CIP	2 (4)		0 (0)	
Against three	ER-STR-CIP	5 (11)	11 (23)	3 (13)	6 (25)
	AMX-ER-NOR	3 (6)		2 (8)	
	AMX-TET-CIP	3 (6)		1 (4)	
Against four	AMX-STR-TET-CIP	3 (6)	3 (6)	1 (4)	1 (4)
Against five	AMX-ER-TET-NOR-AZM	3 (6)	5 (11)	2 (8)	3 (12)
	AMX-ER-TET-NOR-GEN	2 (4)		1 (4)	
Against six	AMX-ER-TET-CIP-NOR-AZM	4 (9)	4 (9)	0 (0)	0 (0)
Total		33 (70)		14 (58)	

*Resistant to antimicrobials at standard doses (μg): amoxicillin (AMX, 30 μg), streptomycin (STR, 10 μg), erythromycin (ER, 30 μg), tetracycline (TET, 30 μg), ciprofloxacin (CIP, 5 μg), norfloxacin (NOR, 10 μg), gentamicin (GM, 10 μg), and azithromycin (AZM, 30 μg)

### Occurrence of antimicrobial residues

Detection of oxytetracycline, ciprofloxacin and enrofloxacin residues by TLC showed their presence in majority of the broiler samples, with higher contamination rate for liver tissues (19 of 24, 79%) than meat samples (16 of 26, 62%). In both kinds of samples, comparatively higher occurrence of oxytetracycline (38 and 31%, respectively) than ciprofloxacin (25 and 19%) and enrofloxacin (17 and 12%) was observed (Table 3).

**Table 3:** Occurrence of antimicrobial residues in meat and liver samples of broiler chickens

Antimicrobial agent	Meat samples (n = 26)				Liver samples (n = 24)			
	Positive [n (%)]	Median ± SD (μg Kg^−1^)	Range (μg Kg^−1^)	Above acceptance [n (%)]	Positive [n (%)]	Median ± SD (μg Kg^− 1^)	Range (μg Kg^− 1^)	Above acceptance [n (%)]
Oxytetracycline	8 (31)	72 ± 52	10–140	2 (8)	9 (38)	86 ± 38	30–155	4 (17)
Ciprofloxacin	5 (19)	93 ± 42	25–130	2 (8)	6 (25)	90 ± 34	50–135	3 (13)
Enrofloxacin	3 (12)	75 ± 29	55–115	1 (4)	4 (17)	81 ± 23	50–105	1 (4)
Total	16 (62)	76 ± 43	10–140	5 (19)	19 (79)	87 ± 33	30–155	8 (33)

UHLP-based quantification of antimicrobial residues in meat and liver samples showed the concentration of oxytetracycline, ciprofloxacin and enrofloxacin ranging from 10 to 155, 25 to 135, and 50 to 115 μg kg^− 1^, respectively (Table 3). Considering the concentration range of these antimicrobials, a significant difference between liver and meat samples was not discernible. However, higher frequency (8 of 24, 33%) of liver than meat (5 of 26, 19%) samples had antimicrobial residues (Table 3) above the recommended level (100 μg kg^− 1^) for food safety [34].

### Environmental health and hygiene practices

**Fig. 3:**
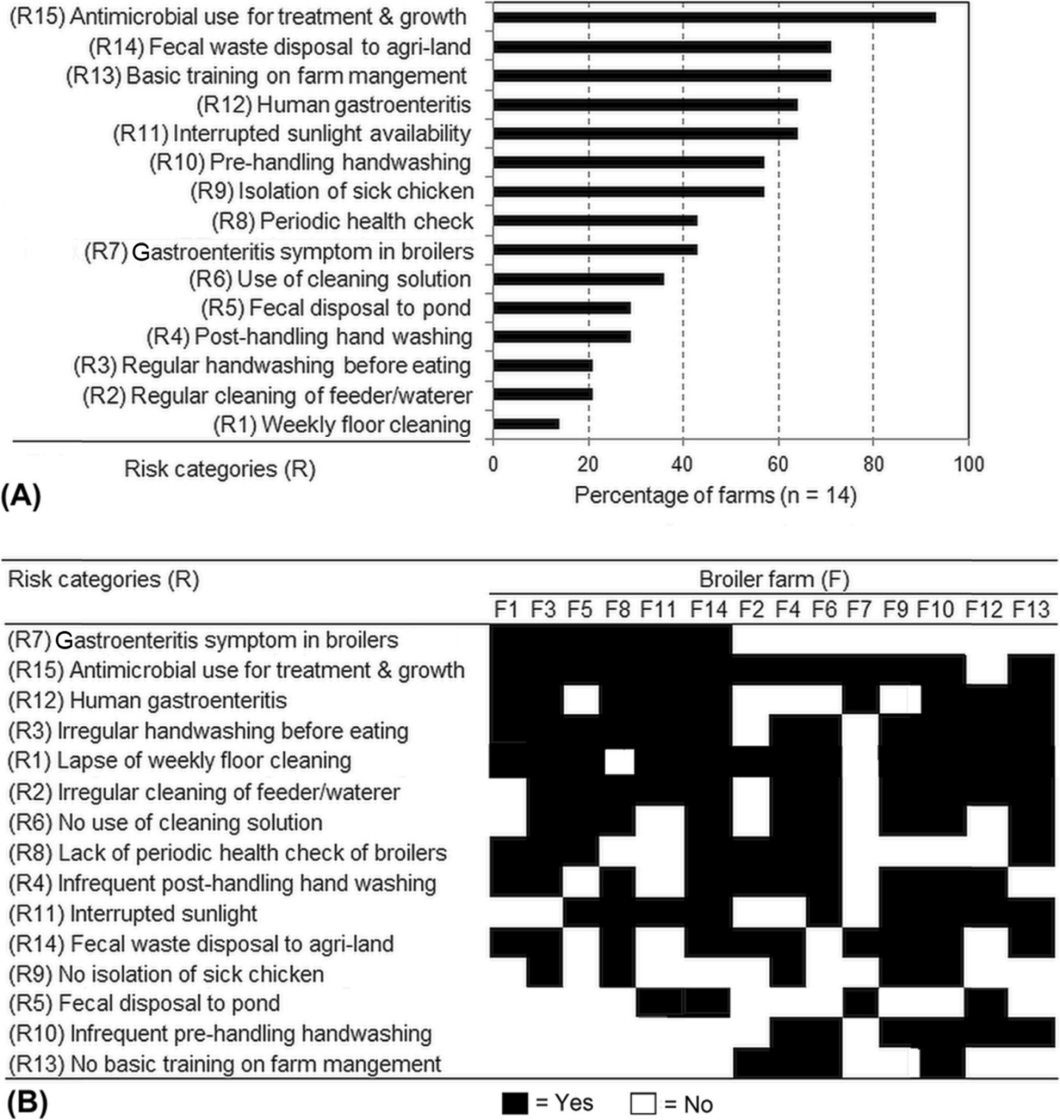
Anthropogenic factors associated with the occurrence of MDR strains of *Campylobacter* in poultry farms. (A) Overall prevalence of the potential risk factors in the selected broiler farms. (B) Individual farm-wise categorization of the risk factors in relation to symptomatic and asymptomatic infections of *Campylobacter*. Data were obtained at two phases (3-month interval) by interviewing (semi-structured interview) a total of 56 representatives from 14 farms, including 9 (F1-F9) screened by laboratory-based microbiological methods to ascertain the contamination sources and prevalent traits of MDR *Campylobacter* spp.

Data obtained from semi-structured interviews of the poultry handlers from the selected 14 broiler farms are summarized in Fig. 3. Among the anthropogenic factors associated with the occurrence of MDR *Campylobacter*, extensive use of antimicrobial agents was captured in all except one of the farms (Fig. 3A). Majority of the farms (71%) usually discarded the poultry-generated waste into agriculture lands while about one-third (30%) of the farms also discarded poultry faeces into aquaculture ponds as fish feed. Quarantine or isolation of sick birds was practiced in 57% of the farms. Periodic health check-up by the veterinary authorities and observation of gastroenteritis were reported for nearly half (45%) of the surveyed farms. Use of disinfectants or cleaning solution to prevent infection from common pathogens and spread of campylobacteriosis, and other gastroenteritis remedies was practiced in approximately one-third (36%) of the farms. On the other hand, regular cleaning of the pots used for feeding and watering, and washing of the floors (of the cages) was done only in small proportion (21 and 14%, respectively) of the farms. Pre-handling hand washing to minimize cross-contamination between broilers was practiced in only 8 out of 14 (57%) farms. However, hand washing interventions, post-handling and before eating, as part of occupational and personal safety, were practiced by only a minor faction (29 and 21%, respectively) of the farmers. All of the representative farmers and poultry-handlers regularly consumed broiler meat and liver reared in their farms. Gastroenteritis among the poultry handlers were reported in majority of the farms, including 5 of 6 farms reporting campylobacteriosis in broilers, which were associated with compound influences of several anthropogenic factors (Fig. 3B). Comparison of symptomatic and asymptomatic infections in individual farms indicated that diverse combinations of multiple risk factors, concerning hygiene (e.g., hand washing, and proper cleaning), poultry rearing practices, waste disposal and health management, were synergistic socio-environmental drivers of the MDR *Campylobacter* pathogens in poultry sector.

## Discussion

Emergence of MDR bacteria from poultry farms is a major threat to public health, especially in low- and middle-income countries. The poultry industry is among the rapidly growing agro-based enterprises with an annual increasing rate of 20% per annum in Bangladesh [35]. Being the major reservoirs of *Campylobacter* spp., poultry and livestock are mainly responsible for the ongoing spread of campylobacteriosis throughout the world. Campylobacters, particularly, *C. jejuni* and *C. coli* have been recognized as the predominant zoonotic bacteria associated with gastrointestinal disorders in humans since the last decade [8, 36]. Recent investigations have claimed a close association of gastroenteritis caused by these pathogens with the widespread occurrence of malnourished under-5 children in developing countries including Bangladesh [6]. The present study provides a glimpse on the occurrence and potential risks of *Campylobacter* pathogens and antimicrobial residues at multiple sources of poultry production and supply chain in Bangladesh.

Studies in tropical regions have observed large differences in *Campylobacter* prevalence in poultry samples, e.g., 32 and 65% of broiler flocks in Vietnam and Ecuador [23, 24]. Similarly, the variations in *Campylobacter* occurrence at farms and LBMs in this study may be attributed to the impacts of differential anthropogenic practices and environmental variations. Although not observed, contaminated chick meconium in hatcheries and also supplied feeds in broiler farms may be a potential source of poultry diseases [37]. Higher occurrence of *Campylobacter* in cloacal swabs is in accordance with the bacterial natural colonization of broiler intestines, considered as the primary source of contamination in poultry products and environment [22]. The predomination of *C. jejuni* over *C. coli* among the isolated strains from both the poultry farms and LBMs has been a common trend worldwide [16, 29, 38]. In comparison to broiler farms, the higher occurrence of *Campylobacter* spp. in meat samples of LBMs is probably linked to large-scale contamination of drinking water and floor environment, facilitating widespread secondary transmission of this zoonotic pathogen. In these LBMs, waters from tube wells or ponds, usually without any treatment, are served in open containers to feed poultry animals, while the maintenance of personal and environmental hygiene is hardly practiced. Even with good farming practices and health interventions, frequent contamination of poultry products by *Campylobacter* spp. at retail markets and slaughter houses is still a major cause of food-borne illness in developed countries in Europe [38, 39].

Large-scale application of antimicrobials as human and veterinary medicine or prophylactic is imposing an immense challenge to public health. In the context of high population density (> 1200 Km^2^) within a small country, the situation is very complex in Bangladesh. High occurrence of MDR strains of *Campylobacter* in the poultry production and supply chain has been reported worldwide [40, 41]. Likewise, a large fraction of *C. jejuni* and *C. coli* populations in poultry samples in this study has been observed as resistant to commonly used antimicrobials, e.g., amoxicillin, erythromycin and tetracycline. Notably, some of these MDR strains, e.g., ca. 20% of *C. jejuni* strains, have also gained resistance to not only β-lactam but also aminoglycoside, quinolone and macrolide, which is in accordance with previous observations [20]. The patterns of resistance traits in *Campylobacter* spp. are usually variable, spatio-temporally and also with geo-socio-climatic variations. This may explain the dissimilarity in major resistance patterns among *Campylobacter* strains reported in different studies. For example, in contrast to the results obtained in this study a low frequency of erythromycin resistant strains but high frequency of resistance to ciprofloxacin, nalidixic acid and tetracycline were reported for *Campylobacter* isolates in Italy and Brazil [10, 36]. The higher frequency of resistant strains in *C. jejuni* than *C. coli,* particularly when considering resistance to multiple antimicrobials, namely, amoxicillin, tetracycline, erythromycin and ciprofloxacin (Table 2, and also see Additional file 4), could be related to the overwhelming natural predominance of the former species. In comparison to *C. jejuni*, higher proportion of *C. coli* strains have been reported to be resistant to tetracycline and erythromycin in Europe and China, respectively [42, 43]. On the other hand, relatively low prevalence of resistant *Campylobacter* strains in market samples than broilers farms (Fig. 2) can be attributable to natural decay of active component of antimicrobials, which are usually applied at the farm level. This inference is also supported by our observation of a significantly (*p* < 0.01) increased abundance of intermediate resistant strains in markets than poultry farms.

The presence of antimicrobial residues, largely unexplored in poultry products, is thought to induce resistance among naturally occurring gut flora and potentially contribute to spreading of MDR strains, eventually health hazards, e.g., gastrointestinal and neurological disorders, hypersensitivity, and tissue damage in animal and human populations [44]. In Ho Chi Minh, Vietnam, the occurrence of ESBL-producing *E. coli* in > 50% of asymptomatic healthy human populations, presumably pre-exposed to residual antimicrobials, coincided with an increasing severity in food-borne diseases [45]. Interaction with antimicrobial chemicals, at sub-lethal concentration, may modulate the bacterial genetic mechanisms conferring MDR traits in *Campylobacter* strains. Among the known genetic mechanisms of such antimicrobial resistance include the horizontal transfer of multiple resistant genes, often found tandemly located in large mobile elements (e.g., plasmids, Class I integron, conjugative transposon) in the bacterial genome and mutations in genes, including *gyrA* and *gyrB* of DNA gyrase and *parC* and *parE* of topoisomerase IV, regulating efflux pump systems and insertional modulation in *cmeB* gene inactivating the CmeABC multidrug efflux pump [46]. The presence of residual content of antimicrobials in most of the tested poultry meat and liver samples in this study is similar to observations reported from another region in Bangladesh [21]. The higher occurrence of such contamination in liver tissue than meat samples (Table 3) is in accordance with their biological processing within poultry animal. In comparison to oxytetracycline, less frequently observed contamination of ciprofloxacin/ enrofloxacin may be related to their relative use in poultry farms. Considering food safety aspects [34], knowledge on the harmful impacts of antimicrobial residues above the acceptance level in broiler tissues, including liver, need to be translated among both the poultry producers and consumers.

Despite sanitation efforts and control measures, in a limited scale, outbreaks of zoonotic diseases are frequent in Bangladesh [47]. According to this study, the level of hygienic and bio-safety measures, e.g., regular hand washing, use of disinfectants, cleaning of the utensils, and washing of the floors and cages are very poor in poultry farms and markets. Therefore, people working in poultry farms and markets are at high risk of occupational health hazards caused by campylobacter infections. An estimated 20–30% of human campylobacteriosis cases have been attributed to imprudent handling, preparation and consumption of broiler meat [36]. Not only the farmers but also other stakeholders, namely, poultry handlers in the LBMs, storekeepers, restaurant owners and household members are responsible to maintain adequate environmental and food hygiene. This study clearly suggests an imperative need to reduce widespread occurrence of secondary contamination of *Campylobacter* in the LBM environment. Adopting optimal slaughtering process with reduced cross-contamination and proper washing with chlorinated water may effectively reduce bacterial loads on chicken carcasses [48]. Results of this study also point out the need of increased efforts in regular health monitoring and quarantine of sick chicken in poultry farms. People residing at a close proximity of poultry farms may also become vulnerable to MDR pathogens because of the large-scale use as fertilizer of poultry-generated waste, which should be properly treated before discarding to adjacent lands and aquatic ecosystems. However, *Campylobacter* infections in a vast majority of people, particularly in developing countries like Bangladesh often remain asymptomatic [6, 13]. The diversity and compound influences of the potential risks of MDR *Campylobacter* (Fig. 3) need to be explored in more details with systematic and long-term observations of their spatio-temporal variations. However, this study did not include investigations measuring the minimum inhibitory compounds, understanding the genetic basis of antimicrobial resistance among the *Campylobacter* isolates, and comparing with antimicrobial resistance patterns among the human populations, which are prospective areas requiring further research. Since antimicrobial-induced genetic recombination and changing patterns in MDR traits in campylobacters poses a major challenge to health interventions, a combined application of multiple strategies, e.g., practicing good husbandry with prudent use of antimicrobials, maintaining adequate hygiene and sanitation, and introducing vaccination, probiotics, prebiotics, antimicrobial peptides, and herbal extracts, may be more effective to promote the sustainable growth of poultry sector [49, 50]. Developing management guidelines to combat zoonotic diseases requires systematic risk assessment along with the dynamics and diversity of MDR pathogens, including campylobacters, which are often challenging due to disproportionate vulnerabilities but crucial to participatory management. In this regard, the variety of sources, contamination level, and differential risks of MDR pathogens in poultry farms and LBMs, and inducing socio-environmental factors identified in this study will facilitate to adopting appropriate interventions to tackle health hazards from campylobacteriosis and other zoonotic diseases.

## Conclusion

The observed high contamination of MDR *C. jejuni* and *C. coli* strains in broiler samples and diverse environmental sources at poultry farms, and their magnified occurrence at LBMs, is obviously related to the coinciding poor status of hygiene, bio-safety, and health management measures, reflecting an alarming situation for food safety in Bangladesh. Moreover, the presence of residual antimicrobials in majority of the broiler liver and meat samples, which may also stimulate MDR occurrence, is an emerging hazard to human and animal health. The systematic approaches followed in this study need to be integrated in future health programs correlating the occurrences of MDR *Campylobacter* in poultry and antimicrobial resistance among human populations, which would provide more insights into the extent of associated socio-environmental hazards. Based on the observed potential risks of MDR *Campylobacter*, it is recommended that not only the farm managers but also more assertively the poultry handlers at markets should be included in behavioural change motivational training programs to adopt preventable measures, including strict maintenance of personal and environmental hygiene, regular monitoring of poultry health and prudent use of antimicrobials. Participatory and holistic surveillance on the transmission dynamics of zoonotic pathogens and adoptable intervention strategies, following ‘One Health’ approaches, need to be promoted through multidisciplinary collaborations; otherwise, the impacts of poultry-originated MDR bacteria and residual antimicrobials to the food chain would bring appalling health disasters in the near future.

## Supplementary information

**Additional file 1. **Primers used for PCR-based detection of the major pathogenic species of *Campylobacter.*

**Additional file 2.** Questionnaire used during ‘Semi Structured Interview’ of poultry farmers or managers.

**Additional file 3. **Representative gel images showing results of genus- and species-specific PCRs of *Campylobacter* isolates.

**Additional file 4. **Antimicrobial resistance patterns of *C. jejuni* and *C. coli* strains from poultry production and supply chain.

## Data Availability

Detail information on the datasets and materials used in this study are available from the corresponding author on reasonable request. This study do not in any way allow poultry farms and markets, respondents, or study communities to be identified. Confidentiality of data is maintained anonymously.

## Abbreviations

AMR: Antimicrobial resistance
BM-M: Broiler meat at LBMs
BM-F: Broiler meat at farms
CDT: Cytolethal distending toxin
CS: Cloacal swab
LBM: Live Bird Market
F: Feed
FS: Floor swab
MDR: Multi-drug resistant
W: Drinking water
TLC: Thin-layer chromatography
UHPLC: Ultra-high performance liquid chromatography
