# Supplementary material for: Risk of multi-drug resistant Campylobacter spp. and residual antimicrobials at poultry farms and live bird markets in Bangladesh
Source: BMC Infect Dis. 2020 Apr 15;20:278. doi: 10.1186/s12879-020-05006-6 (PMC7158023; doi:10.1186/s12879-020-05006-6)
Supplement: Supplementary file 1 — Additional file 1. Primers used for PCR-based detection of the major pathogenic species of Campylobacter. [file 12879_2020_5006_MOESM1_ESM.docx]

**Additional file 1. Primers used for PCR-based detection of the major pathogenic species of *Campylobacter***

| Primer | Sequence (5’-3’) | Target gene | Amplicon | Reference |
| --- | --- | --- | --- | --- |
|  |  |  | size (bp) |  |
| 16S 9F | GAGTTTGATCCTGGCTC | 16S rRNA | 1530 | Samosornsuk et al., 2007 [16] |
| 16S 1540R | AAGGAGGTGATCCAGCC |  |  |  |
| Cj-cdtCU1 | TTTAGCCTTTGCAACTCCTA | *C. jejuni* *cdtC* | 524 | Asakura et al., 2008 [17] |
| Cj-CdtCR2 | AAGGGGTAGCAGCTGTTAA |  |  |  |
| Cc-CdtCU1 | TAGGGATATGCACGCAAAG | *C. coli* *cdtC* | 313 | Asakura et al., 2008 [17] |
| Cc-CdtCR1 | GCTTAATACAGTTACGATAG |  |  |  |
| CfspCU2 | AAGCATAAGTTTTGCAAACG | *C. fetus* *cdtC* | 397 | Asakura et al., 2008 [17] |
| CfspCR1 | GTTTGGATTTTCAAATGTTCC |  |  |  |
