# Supplementary material for: Risk of multi-drug resistant Campylobacter spp. and residual antimicrobials at poultry farms and live bird markets in Bangladesh
Source: BMC Infect Dis. 2020 Apr 15;20:278. doi: 10.1186/s12879-020-05006-6 (PMC7158023; doi:10.1186/s12879-020-05006-6)
Supplement: Supplementary file 2 — Additional file 2. Questionnaire used during ‘Semi Structured Interview’ of poultry farmers or managers. [file 12879_2020_5006_MOESM2_ESM.docx]

**Additional file 2. Questionnaire used during Semi Structured Interview** **of poultry farmers or managers**

**Questionnaire: Farm Management and Socio-environmental Risk Factors**

**Project: Improving Food Safety in Bangladesh**

Conducted by: Department of Microbiology and Hygiene, Bangladesh Agricultural University

Supported by: Department of Livestock Services (DLS) and Food Safety Program (FSP-UN), Bangladesh

Form ID ____________ Interview phase (1/2)_______ Team (A/B)_________

Date _______­­­__ Location___________ Poultry farm code_______________________

**A. General Information**

1. Designation of key informant______________________________________

2. Address code: __________________________________________________________

3. Primary source of income

Poultry____ Poultry-associated______ Others, explain___________

4. Establishment year of poultry farm: ______

5. Production rate (per batch)

Highest yield: __________________ Lowest yield: _______________________

6. Poultry housing

Area (m2): _______ Newly built _______ Not renovated_________

7. Distance and number of nearby houses

Distance (m) of the nearest house _____ Number of houses within (1 km^2^) _______

**B. Knowledge and Rearing Practices**

8. Number of handlers

Direct_________ Indirect/Occasional handlers__________

9. Training/certification of the farmer or workers

No_______ Have training_______ Type, duration______________________

10. Type(s) of feed used

Type(s)___________________ Prepared at farm ______ Purchased_________

11. Use of prophylactic supplements

No _________ Yes__________ Kind__________

12. Source and treatment of drinking water

Shallow well water___ Deep tube well water_____ Tap water_____ Pond/river water______

No water treatment_____ Water treatment before supply as drinking water__________

13. Water quality examination?

No___ Yes, frequency _______

14. Use of antibiotics in the past month(s)

Yes________ No___________

For what purpose? ___________________________________________________

15. Periodic health check

No ___ Yes______ By whom _______________________

16. Campylobacteriosis/similar infections:

No knowledge_____ Have basic understanding ______________

Can differentiate from other enteric diseases_______

17. Number of animals suffering enteric infection per batch: _______

Treatment/ therapy: _________________

18. Isolation of the sick from healthy animals

No__________ Yes____________ Where & how long_____________________

**C. Farm Environment & Hygiene Conditions**

19. Floor cleaning

Regularly________ Weekly _______ Monthly______ No cleaning per batch_______

20. Cleaning of cages with fecal matter,

Regularly________ Weekly _______ Monthly______ No cleaning per batch_______

21. Water container cleaning

Regularly________ Weekly _______ Monthly______ No cleaning per batch_______

22. Feeding container cleaned before using?

Regularly________ Weekly _______ Monthly______ No cleaning per batch_______

23. Washing hands, before handling/feeding:

Always ________ Irregularly_______ No practice__________

24. Presence/visit of other animals (wild or domestic)

No_____ Yes_______ What type(s)______________ How frequently ______________

25. Condition of poultry sheds

Wet all seasons__________ Wet during particular season___________

Any special protection of dry, shaded area? ___________

26. Sunlight accessibility

High___________ Medium____________ Poor____________

27. Do poultry animals roam and feed from the yard or stay in the farm?

Feed in fenced yard _______ Always in poultry shed _______ Other ______________

28. Type of cleaning agents

Untreated water ___ Treated water______ Cleaning agent- what? ____________________

29. Fecal waste management

Discharged directly to ponds_____ Used as fertilizer _______ Other, explain__________

30. Observation of gastroenteritis/diarrhea symptom

No_________ Yes__________ Frequency ________

**D. Caretaker’s Health & Hygiene**

30. Does caretaker/other live within the farm premise?

No ___ Yes, number______

31. Does the farmer consume his/her own production?

Yes ______ No_________

32. Occurrence of gastroenteritis/diarrhea

No__________ Yes, frequency_________

33. If sick, do the caretakers still carry out farm handling?

No ______ Yes, always ______ Sometimes_______

34. Hand washing practice

Before eating or cooking_____ After using toilet______ After handling raw meat_____

35. Use of soap

No_________ Always________ Sometimes _________

36. Separation of raw meat from fruits/vegetables in the kitchen?

No___ Yes___

**Other comment(s) /observation(s) on risk factors (if any)**

**Verification of data**

Collected by: Verified by:

____________________________________ ________________________________

Name, Signature & Date Name, Signature & Date
