# Supplementary material for: Risk of multi-drug resistant Campylobacter spp. and residual antimicrobials at poultry farms and live bird markets in Bangladesh
Source: BMC Infect Dis. 2020 Apr 15;20:278. doi: 10.1186/s12879-020-05006-6 (PMC7158023; doi:10.1186/s12879-020-05006-6)
Supplement: Supplementary file 3 — Additional file 3. Representative gel images showing results of genus- and species-specific PCRs of Campylobacter isolates. [file 12879_2020_5006_MOESM3_ESM.docx]

**Additional file 3. Representative gel images showing results of genus- and species-specific PCRs of *Campylobacter* isolates**

**
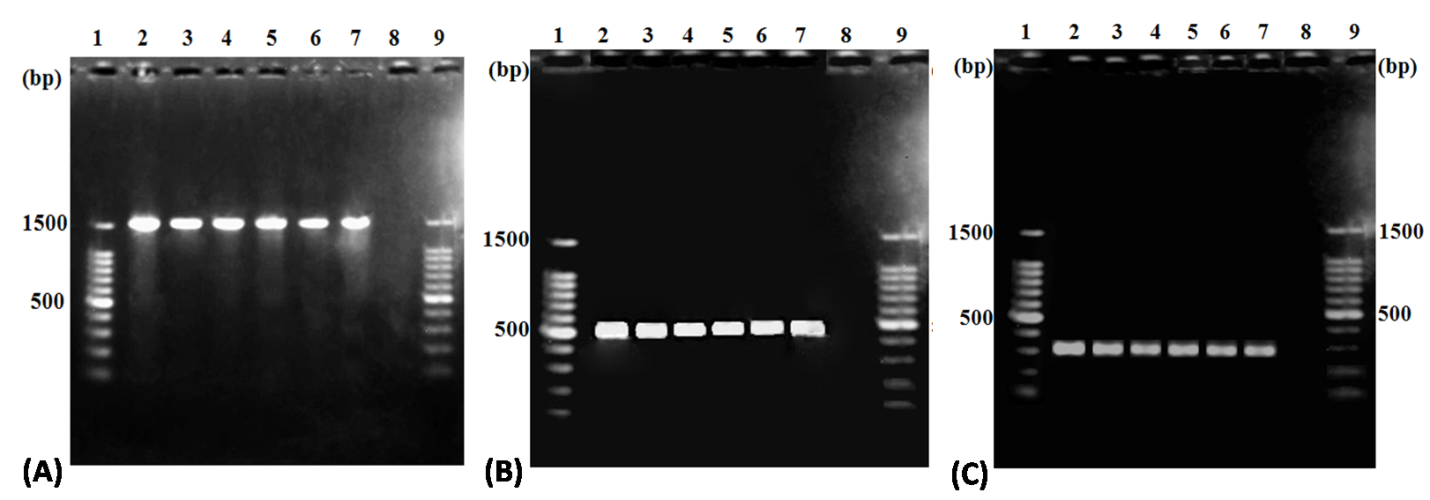
**

Figures showing (A) 16S rRNA gene-based PCR to identify *Campylobacter* genus (B) *cdtC* gene-based multiplex PCR assay to identify *C. jejuni*, and (C) *cdtC* gene-based multiplex PCR assay to identify *C. coli*. In all figures, lanes: 1 and 9, 100 bp DNA ladder (Promega, USA); lanes 2 and 8, positive and negative controls, respectively; and lanes 3-7, representative positive isolates obtained from culture-based methods.
