# Supplementary material for: Risk of multi-drug resistant Campylobacter spp. and residual antimicrobials at poultry farms and live bird markets in Bangladesh
Source: BMC Infect Dis. 2020 Apr 15;20:278. doi: 10.1186/s12879-020-05006-6 (PMC7158023; doi:10.1186/s12879-020-05006-6)
Supplement: Supplementary file 4 — Additional file 4. Antimicrobial resistance patterns of C. jejuni and C. coli strains from poultry production and supply chain. [file 12879_2020_5006_MOESM4_ESM.docx]

**Additional file 4. Antimicrobial resistance patterns of *C. jejuni* and *C. coli* strains from poultry production and supply chain**

| Antimicrobial agent | *C. jejuni* (n = 47) | | |  | *C. coli* (n = 24) | | |
| --- | --- | --- | --- | --- | --- | --- | --- |
| (Standard dose, μg) | Resistant (%) | Intermediate (%) | Susceptible (%) |  | Resistant (%) | Intermediate (%) | Susceptible (%) |
| Amoxicillin (30 μg) | 30 (64) | 12 (25) | 5 (11) |  | 13 (54) | 7 (29) | 4 (17) |
| Tetracycline (30 μg) | 29 (62) | 8 (17) | 10 (21) |  | 11 (46) | 6 (25) | 7 (29) |
| Erythromycin (30 μg) | 24 (51) | 13 (28) | 10 (21) |  | 10 (42) | 6 (25) | 8 (33) |
| Ciprofloxacin (30 μg) | 17 (36) | 9 (19) | 21 (45) |  | 7 (29) | 8 (33) | 9 (38) |
| Norfloxacin (10 μg) | 12 (26) | 8 (17) | 27 (57) |  | 8 (33) | 6 (25) | 10 (42) |
| Streptomycin (10 μg) | 12 (26) | 6 (13) | 29 (62) |  | 6 (25) | 2 (8) | 16 (67) |
| Azithromycin (30 μg) | 7 (15) | 6 (13) | 34 (72) |  | 4 (17) | 5 (21) | 15 (62) |
| Gentamicin (10 μg) | 2 (4) | 4 (9) | 41 (87) |  | 2 (8) | 3 (13) | 19 (79) |
